# Factors of Caregiving Resilience by Race-Ethnicity in a National Sample of Caregivers

**DOI:** 10.1093/geroni/igab046.205

**Published:** 2021-12-17

**Authors:** Jinmyoung Cho, Natasha Peterson

**Affiliations:** 1 Baylor Scott & White Health Research Institute, Temple, Texas, United States; 2 Iowa State University, Iowa State University, Iowa, United States

## Abstract

Despite heavy burdens and responsibilities, some caregivers are more likely to cope better with their care responsibilities than others, and this could vary by cultural beliefs and norms on caregiving. This study examined contributing factors of resilience with three racial-ethnic groups (White, Blacks, Hispanic). A total of 2,652 caregivers were included from Round 7 of the National Study of Caregiving. Caregiving resilience was defined by higher levels of care demands and higher levels of psychological well-being. Five domains of contributing factors were included: socio-demographic characteristics, context of care, caregivers’ psychological attributes, informal and formal support. Multiple logistic regressions showed that caregivers with higher psychological attribute levels were more likely to be resilient in all three groups. However, unique predictors have also been observed by race-ethnic groups (e.g., Blacks using formal support were more resilient). These findings suggest the need for culturally specific programs to facilitate resilience among caregivers.

